# Responses of Soil Microbial Communities and Networks to Precipitation Change in a Typical Steppe Ecosystem of the Loess Plateau

**DOI:** 10.3390/microorganisms10040817

**Published:** 2022-04-14

**Authors:** Yutao Wang, Yingzhong Xie, Hongbin Ma, Yi Zhang, Juan Zhang, Hao Zhang, Xu Luo, Jianping Li

**Affiliations:** College of Agriculture, Ningxia University, Yinchuan 750021, China; yutaowang@nxu.edu.cn (Y.W.); xieyz@nxu.edu.cn (Y.X.); ma_hb@nxu.edu.cn (H.M.); 13995378736@163.com (Y.Z.); 18435121531@163.com (J.Z.); 82101211051@caas.cn (H.Z.); luoxu181226@163.com (X.L.)

**Keywords:** microbial community structure, precipitation gradients, networks, Loess Plateau

## Abstract

The response of microbial communities to changes in precipitation can regulate the nutrition cycling of terrestrial ecosystems, but the effect on the structure and interaction of microbial communities and the relationship with environmental factors in arid and semiarid areas are unclear. Here, a field simulation experiment using three precipitation gradients, 50% of normal precipitation (P50), normal precipitation (P100) and 150% of normal precipitation (P150), was carried out in the typical grassland of the Loess Plateau. We applied high-throughput sequencing and network analysis to explore the effect of precipitation changes to soil microbial communities. The results indicated that the structural composition of the microbial community responded to precipitation treatments dramatically. The Top 50 microbials were divided into resource-limited, drought-tolerant and sensitive groups based on their response to altered precipitation. The network of bacteria was more complex and stronger than fungi. Bacterial networks were less stable but more adaptable under drought than fungal. Increasing precipitation promoted the complication and firmness of fungi networks. These findings are crucial for revealing the effects of climate change on soil microbial communities in arid-land and elsewhere and can provide valuable guidance for ecological restoration and response to climate change of the Loess Plateau.

## 1. Introduction

The exacerbated hydrological cycle and water shortage caused by global climate change and the resulting deep impact are rapidly becoming serious climate and environmental problems on a global scale [1,2], especially in fragile arid and semiarid ecosystems [3]. Studies have shown that the vast areas of western China will be even drier under a temperature rise level of 1.5–2 °C [4]. The temporal and spatial distribution of available water is the main driving force of biogeochemical cycles in arid and semiarid ecosystems [5,6]. Therefore, understanding the response mechanisms of arid and semiarid ecosystems to changes in precipitation is crucial to their management and development.

The soil microorganism is one of the most important participants in the nutrient cycling process of the terrestrial ecosystem [7,8,9], such as the decomposition of soil organic matter, the circulation of soil nutrients and the stoichiometry of plants, which play a key driving role in the biogeochemical cycle [10]. Research on the response of soil microbial communities to altered precipitation can greatly improve the predictive ability of ecosystems in response to global climate change due to their important regulatory effects on terrestrial ecosystems [1,11].

Precipitation pattern changes, such as the scale, frequency and duration of precipitation, will cause a threat to fragile terrestrial ecosystems with the dynamic changes in soil moisture [12], especially in arid and semiarid-land [13]. Water is a limiting factor for ecosystems in arid and semiarid areas and an important substance for microbial metabolism, which transports the medium for solutes and microorganisms [14]. To date, there have been many studies on the response of soil microorganisms to altered precipitation in some specific ecological environments. Ren used meta-analyses to study the responses of soil microbial characteristics like biomass and community composition to altered precipitation on a global scale and showed that precipitation treatments had a significant impact on total soil microbial biomass but a small impact on community composition [8]. The abundance of bacteria was more sensitive than that of fungi under drought conditions, and it was mainly affected by the scale of precipitation rather than the duration. Conversely, a study on grasslands on the Qinghai-Tibet Plateau showed that altered precipitation significantly affected the diversity and community structure of microorganisms [15]. Some studies considered that fungi were more sensitive to changes in precipitation than bacteria in vegetation ecosystems [16,17]. Others believed that altered precipitation does not change the community structure of soil microbes in grassland ecosystems [18,19]. Studies in high-desert grasslands considered that the impact of precipitation on the microbial community was limited [20]. Investigations on the Loess Plateau have shown that extreme precipitation events have significantly affected the diversity and abundance of soil bacterial and fungal community composition [21,22]. Abbasi’s meta-analysis indicated that bacterial and fungal communities were resistant to precipitation reduction [14]. Based on existing research, there are still unclear and diverse responses to the diversity, community structure and interactions of microorganisms to changes in precipitation [23]. Determining the response process of soil microbes to precipitation treatments has theoretical and practical significance for predicting the responses of soil microbes in the Loess Plateau to future global climate changes.

Changes in precipitation can affect the network of microorganisms [15,24]. The robustness of the microbial network increases with increased precipitation, while it is unstable and fragile in an extremely dry environment [24]. Decreased precipitation may promote the separation of bacteria and reduce competition between bacterial species [25]. The analysis of the network of microorganisms under precipitation changes is a new perspective aimed at studying the response of microbial communities to altered precipitation, and it also provides information for the feedback mechanism of soil microorganisms to changes in precipitation [26].

Soil microorganisms do not live in isolation but are closely related to environmental factors, forming a complex ecological interaction network [27]. Factors affecting soil microbes include soil pH and decomposition of soil organic matter [22], average annual precipitation [15,28], and plant community composition [15,29]. The magnitude and duration of the impact of precipitation on microorganisms are affected by the inherent temporal and spatial heterogeneity, which results in differences in the available soil resources [5]. Therefore, it is inevitable to consider the soil-vegetation-microbial relationship when considering the response of the soil microbial community to altered precipitation.

The Loess Plateau is a typical ecologically fragile area that plays an important role in the ecosystems of China [30]. Our research mainly investigated the response of environmental factors, microbial diversity, community structure and microorganism interactions on the Loess Plateau after two years of simulated precipitation. The study was undertaken mainly to improve the response mechanism of microbes to changes in precipitation in arid and semiarid grassland ecosystems and to promote our understanding of the potential response of fragile terrestrial ecosystems to global precipitation changes and the services they provide. We propose the following hypotheses: (1) soil bacterial and fungal diversity following precipitation grades adapted to changing soil moisture and vegetation; (2) bacteria and fungi interconnected patterns across treatments is different; (3) The network stability of soil bacteria is poorer than fungal ones under decreased precipitation.

## 2. Materials and Methods 

### 2.1. Study Area

The experiment was carried out in Yunwu Mountain on the Loess Plateau in Guyuan city, Ningxia Province, northwestern China (106°21′ E–106°27′ E, 36°10′ N–36°17′ N), with an elevation of 1700–2148 m and a slope of 7–10°. The typical temperate continental semiarid monsoon climate has an annual average precipitation of 439 mm, which mostly occurs from June through September, and the annual average temperature is 7.2 °C. Meteorological data based on the area in the past 30 years were obtained. The main vegetation types are *Stipa bungeana*, *Artemisia gmelinii*, *Stipa grandis*, *Artemisia frigida*, *Potentilla acaulis* and *Agropyron michnoi* [31].

### 2.2. Experimental Design and Sample Collection and Processing

**Figure 1:**
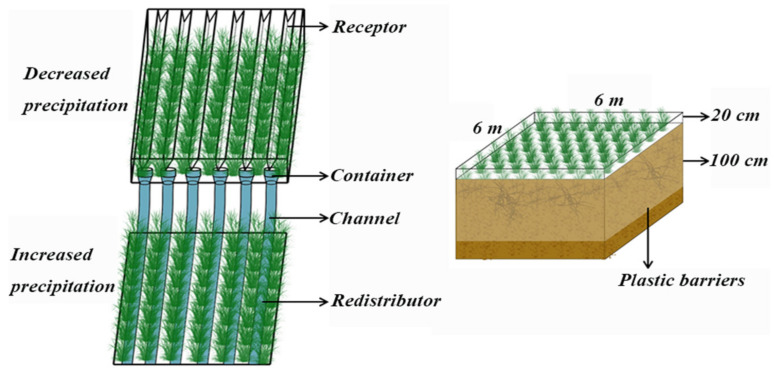
Rain shelter and irrigation devices in the study area.

The field simulation precipitation device was established in July 2017. According to the analysis of the meteorological data of the study area for the past 30 years, the precipitation variation range of the study area was approximately 50% of the annual average precipitation, with the minimum value being 282 mm in 1982 and the maximum being 706 mm in 2013. To allow the experimental research to offer practical predictions and guiding significance for precipitation grades in the near future, the simulation device was set to three precipitation gradients, namely, normal precipitation (P100), 50% of normal precipitation (P50) and 150% of normal precipitation (P150) (Figure 1). To avoid differences in soil properties caused by spatial heterogeneity, the study plots were all selected on uniform grassland. Nine 6 m × 6 m plots (three gradients × three replicates, replicates = blocks) were randomly set with 2 m buffer zones to avoid marginal effects. A water interception-catchment system was used to irrigate 50% of the precipitation intercepted by the canopy to the adjacent neighborhood to form a precipitation reduction area (P50) and a precipitation enhancement area (P150). The canopy adopted an open design using 2 mm thick transparent polyethylene material, and its light transmittance reached more than 95%. The polyethylene panels were placed on an iron frame fixed on the ground to achieve the experimental purposes of shielding precipitation and maximum ventilation at the same time. The intercepted precipitation flowed into the corresponding water collection trough through the groove of the V-shaped interception plate and then irrigated evenly in the increased precipitation area (P150). A 120 cm plastic water barrier (10 cm above ground and 110 cm underground) was set around each plot to prevent surface runoff and groundwater infiltration.

Soil and plant samples were collected in July 2019. A soil auger was used to collect 0–30 cm soil with 10 cm intervals; at each plot, three duplicate soil samples were dug at each soil layer, and then the same layers of soil were mixed into a sample and the litter and impurities were eliminated through a 2 mm sieve, producing a total of 27 soil samples. All samples were divided into two parts: one was naturally air-dried for soil property analysis and the other was immediately frozen at −80 °C for high-throughput sequencing.

### 2.3. Plant and Soil Physicochemical Characteristic Analyses

A 1 m × 1 m square from each plot was randomly selected to investigate the vegetation diversity, and biomass was harvested aboveground, litter, and belowground; plant samples were cleaned and then put in an oven at 80 °C for 48 h to obtain their biomass value. All of the living and dead aboveground biomass in the square was cut together, the dead part was removed and combined into litter biomass, and the living biomass was the aboveground biomass. The underground biomass was measured for all plant roots within 0–30 cm soil. A digital pH meter (PHS-3C; Shanghai) was used to measure soil pH at a 1:2.5 (*w/v*) soil-to-water ratio. Fresh soil samples were oven-dried at 105 °C for 48 h to get the soil moisture content. Soil organic carbon (C) was determined by the potassium dichromate titration method [32]. Soil total nitrogen (N) was measured by micro-Kjeldahl digestion (Elementar, Vario EL III, Langenselbold, Germany) [33]. The total phosphorus (P) and available phosphorus (AP) concentrations were determined by molybdenum blue colorimetry [34]. Soil means weight diameter (MWD), soil fractal dimension (SFD) and soil geometer mean diameter (GMD) refer to Le Bissonnais’s rapid wetting standard method to measure the stability of soil aggregates [35]. Soil compactness was determined by a digital display soil compaction meter (SC900 soil compaction meter, Spectrum Technologies Inc., Aurora, IL, USA).

### 2.4. Soil DNA Extraction and Gene Sequencing

After extracting soil microbial DNA using the E.Z.N.A.^®^ Soil DNA Kit (Omega Biotek, Norcross, GA, USA), the DNA extraction quality was checked by 1% agarose gel electrophoresis, and DNA concentration and purity were determined using a NanoDrop2000 UV-vis spectrophotometer (Thermo Scientific, Wilmington, DE, USA). The bacterial 16S rRNA gene V3-V4 hypervariable region and fungal ITS hypervariable region of soil was amplified by PCR using an ABI GeneAmp^®^ 9700 PCR thermocycler (ABI, Vernon, CA, USA). The primer pairs for bacteria and fungi were 338F_806R (5′-ACTCCTACGGGAGGCAGCAG-3′_5’-GGACTACHVGGGTWTCTAAT-3’) and ITS1F_ITS2R (5′-CTTGGTCATTTAGAGGAAGTAA-3′_5′-GCTGCGTTCTTCATCGATGC-3′), respectively. The polymerase in the PCR process for bacterial/fungal DNA was TransStart Fastpfu DNA Polymerase/TaKaRa rTaq DNA polymerase. Twenty-microliter bacteria/fungi mixtures for PCR contained 4 μL of 5× TransStart FastPfu buffer/2 μL of 10× Buffer, 2.5 mM dNTPs in 2 μL, 0.8 μL of forward primer (5 μM), 0.8 μL of reverse primer (5 μM), 0.4 μL of FastPfu Polymerase/0.2 μL of rTaq Polymerase, 0.2 μL of BSA, 10 ng of template DNA, and finally ddH2O up to 20 μL, with 3 replicates per sample. The PCR parameters were the initial denaturation performed at 95 °C for 3 min, annealing at 55 °C for 30 s, elongation at 72 °C for 45 s, and final extension at 72 °C for 10 min, and the reaction was halted at 10 °C. After mixing the PCR products of the same sample, an AxyPrep DNA Gel Extraction Kit (Axygen Biosciences, Union City, CA, USA) was used to purify the products, 2% agarose gel electrophoresis was used to detect the products, and the quantification of product was detected by a Quantus™ Fluorometer (Promega, Madison, WI, USA). The NEXTFLEX Rapid DNA-Seq Kit was used for library construction, and Illumina’s MiSeq PE300 platform was used for sequencing (Shanghai Majorbio Biopharm Technology Co., Ltd., Shanghai, China). Trimmomatic was used for quality control of the original sequence, FLASH was used for splicing, and Uparse was used to determine operational taxonomic units (OTUs) based on a 97% similarity cluster and to eliminate chimeras [36]. Using the Ribosomal Database Project (RDP) classifier to classify and annotate each sequence [37], bacteria and fungi were compared with the Silva and Unite database, respectively [38,39], and the comparison threshold was set to 70%. All subsequent analysis of data from the samples adopted the minimum sequence number of OTUs.

### 2.5. Statistical Analyses

Variance in environmental factors and microbial alpha diversity were determined using SPSS 22 (SPSS Inc., Chicago, IL, USA) to detect differences among different treatments, and Origin2021 (Origin Lab 2017, Northampton, MA, USA) was used to draw histogram figures. Leveled OTU data were used to analyze the community composition of bacteria and fungi and to count the types and proportions of microorganisms contained in each sample at each level of classification. A generalized linear mixed model (GLMM) was used to analyze the effect of altered precipitation on microbial species by SPSS; the fixed and nested factors were precipitation changes and soil layer, respectively [13]. The statistical calculation of OTUs was performed using Usearch 7 [40]. The alpha diversity of microbes was analyzed by R (Veen Diagram package). The OTU Venn analysis of biological communities under different treatments and the statistical analyses of unique and common OTUs were performed using Mothur 1.30.2 [41]. The beta diversity distance was calculated using QIIME 1.9.1. Principal coordinate analysis (PCoA) was applied to distinguish the community structure of microbes under different treatments with the Meiji biological cloud platform, which was based on the Bray-Curtis distance, with OTUs as the species classification level, and the analysis of differences between groups adopted Adonis. Cytoscape 3.5.1 was used to draw a network diagram and analyze the interaction types of microorganisms [42]. Only the Top 50 total abundance of OTUs was selected, with a correlation *p* value < 0.05 indicating significant correlation by Spearman correlation analysis. VIF (variance inflation factor) was used to screen environmental factors and retain environmental factors with less collinearity, and then Canoco 5.0 and R were used to perform redundancy analysis and generate a heatmap [30,43] to analyze the relationship between the microbial community and environmental factors.

## 3. Results

### 3.1. Soil and Vegetation Characteristic under Precipitation Treatments

The diversity index and richness index of vegetation decreased significantly in P50 (*p* < 0.05), but an insignificant reduction in P150 occurred. The vegetation coverage was highest in P150, while it was lowest in P50. The above-ground biomass of vegetation increased with increasing precipitation. Compared to P100, the above-ground biomass was increased by 13% in P150 but decreased by 38% in P50. Litter biomass had an insignificant response to altered precipitation but was the largest in P50. Increased and decreased precipitation significantly reduced the underground biomass of vegetation by 52% and 65%, respectively (Table 1).

Soil moisture was significantly influenced by the interaction of soil layers and precipitation (*p* < 0.05) (Table 2). Soil organic carbon and available phosphorus decreased with increasing soil depth, but soil pH was positively correlated to soil depth (*p* < 0.05). P50 and P150 decreased soil pH and improved soil compactness, and the soil water content increased with increasing precipitation (*p* < 0.05).

### 3.2. Diversity of Soil Microbial Communities under Precipitation Treatments

#### 3.2.1. Venn Diagram of Microorganisms under Precipitation Treatments

**Figure 2:**
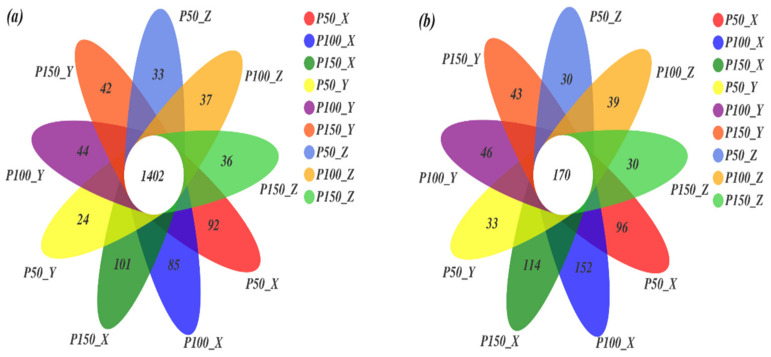
Venn diagrams of bacteria (**a**) and fungi (**b**) show the common and unique OTUs under different treatments. X: 0–10 cm; Y: 10–20 cm; Z: 20–30 cm. OTUs = Operational Taxonomic Units. The same as below.

The total bacteria/fungi in the soil sample had 1,254,589/1,741,654 effective sequences, 5494/2316 OTUs, 35/10 phyla and 1292/522 species, and the average effective sequence length was 419.37/232.52 bp. The rarefaction curve tended to be flat, which means species are evenly distributed in the sample, and the amount of sequencing data was reasonable, which can reflect most of the microbial diversity information in the sample. The OTU table was normalized before calculating the richness. The OTUs of bacteria and fungi under different treatments are shown in the Venn diagram (Figure 2). The numbers of bacterial and fungal common OTUs were 1402 and 170, respectively. Compared with P100, P50 and P150 reduced the unique OTUs of bacteria and fungi, except for bacteria at 0–10 cm, which showed a completely opposite increasing trend in both P50 and P150. The largest variation in unique microbial OTUs was observed at P50. The number of unique OTUs of bacteria and fungi decreased as the soil layer deepened, and the variation in soil microorganisms affected by altered precipitation decreased as the soil layer deepened. The number of unique bacterial and fungal OTUs accounted for 9% and 25% of the total OTUs under different treatments, respectively.

#### 3.2.2. Diversity of Microbes under Different Precipitation Treatments

**Figure 3:**
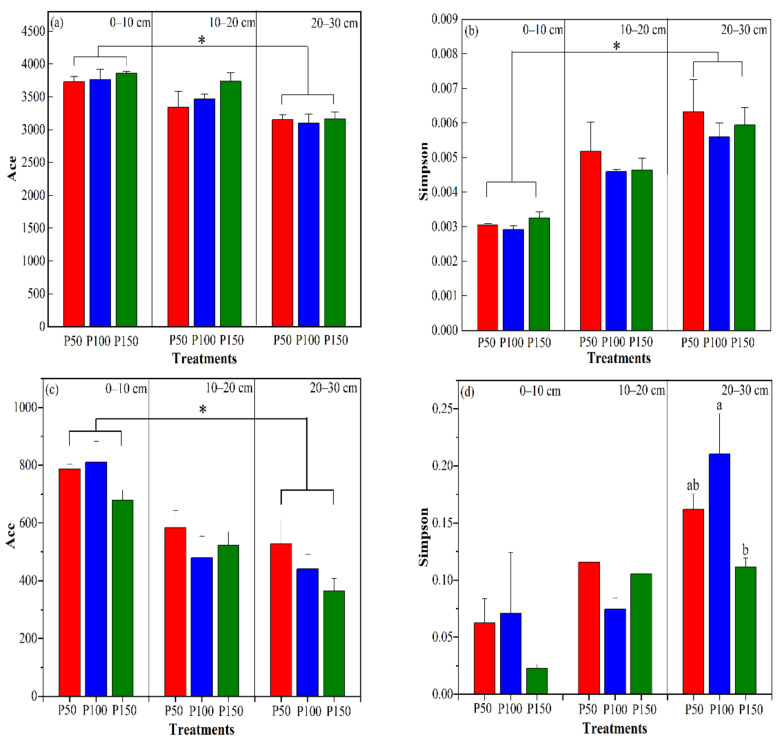
The dynamics of the microbial α-diversity index under different precipitation treatments and soil layers. The bacterial and fungal richness was measured by the Ace index in (**a**,**c**), respectively; the bacterial and fungal α-diversity was measured by the Simpson index in (**b,d**), respectively. Different lowercase letters indicate significant differences at 0.05 (*p* < 0.05) among precipitation treatments. * indicate *p* < 0.05 among different soil layer.

Precipitation treatments had no effects on bacterial community richness (Ace, Figure 3a) or α-diversity (Simpson, Figure 3b). Bacterial richness was insignificantly reduced under altered precipitation and varied greatly in P50. The richness and α-diversity of bacteria decreased as the soil layer deepened, and the highest values occurred in the surface layer (*p* < 0.05).

The richness of fungi did not differ significantly under altered precipitation (Figure 3c). The diversity of fungi was significantly different at 20–30 cm, increasing by 47% in P150 and 23% in P50 (Figure 3d). Compared with the control, the richness of fungi decreased by 16% in P150 and 3% in P50 in the surface layer (0–10 cm), while the diversity of fungi increased by 68% in P150 and 12% in P50 at 0–10 cm, respectively. The fungal communities were more sensitive to P150. The Simpson index of fungi was significantly greater in the surface layer (*p* < 0.05).

### 3.3. Composition of Soil Microbial Communities under Precipitation Treatments

**Figure 4:**
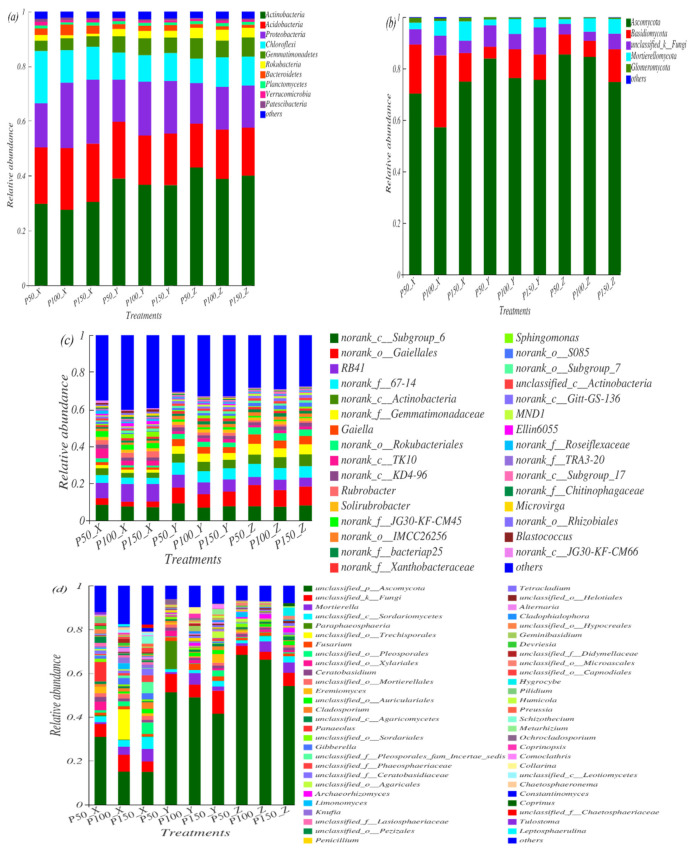
The relative abundance of soil bacterial/fungal communities at the phylum (**a**,**b**) and genus (**c**,**d**) levels under different treatments. Others < 0.01.

At the phylum level, the main bacterial communities were Actinobacteria, Acidobacteria, Proteobacteria, Chloroflexi, Gemmatimonadetes, Rokubacteria and Bacteroidetes (others < 0.01), which collectively accounted for 93.8–95.4% of all taxon sequences (Figure 4a). The *p* value of the omnibus results by GLMM showed that Actinobacteria, Proteobacteria, Gemmatimonadetes, Rokubacteria, and Bacteroidetes were significantly different (*p* < 0.05). The relative abundance of Actinobacteria increased in P50 (8%) and P150 (4%), while the relative abundances of Proteobacteria and Bacteroidetes decreased at P50 and P150. The relative abundance of Proteobacteria decreased by 22% in P50 and 2% in P150. The relative abundance of Bacteroidetes decreased by 40% and 24% in P50 and P150 at the surface layer (0–10 cm), respectively. The relative abundances of Gemmatimonadetes and Rokubacteria increased in P50 but decreased in P150. The relative abundance of Rokubacteria increased by 89% in P50 but decreased by 12% in P150 at the surface layer.

At the phylum level, the dominant fungal community were (Others < 0.01) Ascomycota, whose relative abundance in all samples was approximately 57.26–85.5%, followed by Basidiomycota, unclassified_k__Fungi, Mortierellomycota and Glomeromycota (Figure 4b). The GLMM set the precipitation treatments as the main influencing factor, the soil layer was the nested factor, and the *p* value of the omnibus results showed that Ascomycota, Basidiomycota, Mortierellomycota and Glomeromycota were significantly different among treatments (*p* < 0.05). The relative abundance of Ascomycota and Basidiomycetes increased with increasing soil depth under P50; the relative abundance of Ascomycota increased but Basidiomycetes decreased, with increasing soil depth under P150. The relative abundances of Ascomycota increased in P50 (10%) and P150 (3%); the relative abundances of Glomeromycota increased by 67% in P50 and 22% P150; the relative abundances of Basidiomycetes decreased under P50 (31%) and P150 (26%); the relative abundances of Mortierellomycota decreased by 59% and 0.36% in P50 and P150, respectively.

At the genus level, GLMM results showed that the top 20 bacterial species with significant differences under different precipitation conditions were Gaiellales, RB41, 67-14, Actinobacteria, Gemmatimonadaceae, Gaiella, Rokubacteriales, Rubrobacter, JG30-KF-CM45, IMCC26256, bacteriap25, Sphingomonas, S085 and MND1 (*p* < 0.05) (Figure 4c). The relative abundances of Rubrobacter, Sphingomonas, Ellin6055, and Microvirga increased by 29%, 15%, 11% and 15% in P150, respectively, while 67–14, Gemmatimonadaceae, Gaiella and Rokubacteriales increased by 13%, 7%, 16% and 10% in P50, respectively. The relative abundances of RB41, IMCC26256, and bacteriap25 were the highest in the control, while Gaiellales, Actinobacteria, and Gaiella were the lowest in the control.

At the genus level, GLMM results showed that the top 20 fungal species with significant differences under different precipitation conditions were Ascomycota, Mortierella, Pleosporales, Gibberella, Incertae_sedis, Phaeosphaeriaceae, Ceratobasidiaceae, Archaeorhizomyces, Knufia, Lasiosphaeriaceae, Pezizales, Penicillium, Tetracladium, Geminibasidium and Devriesia (Figure 4d). Compared with P100, the relative abundance of Pleosporales, Incertae_sedis, and Phaeosphaeriaceae increased by 3 times, 4.7 times and 3 times in P150, respectively. However, Ascomycota, Pezizales, and Devriesia increased by 16%, 167%, and 35% in P50, respectively. The relative abundances of Knufia and Geminibasidium were the highest in the control, but Ceratobasidiaceae had the lowest value in the control.

### 3.4. Microbial β-Diversity Analysis under Treatments

**Figure 5:**
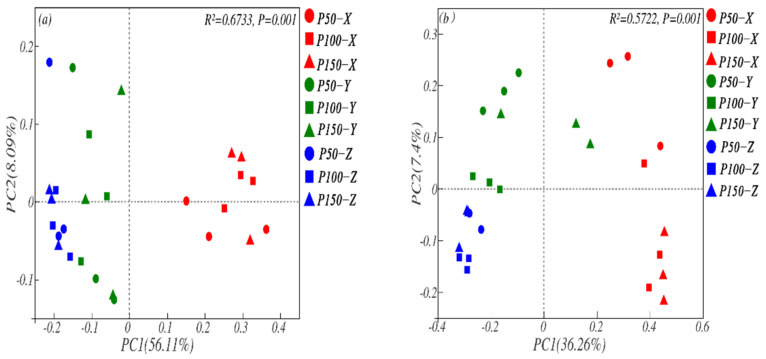
PCoA of bacterial (**a**) and fungal (**b**) communities at the OUT level under different treatments. R^2^ and *p* value represents the difference between different samples of treatments. The closer R^2^ is to 1, the more obvious the difference between samples. *p* < 0.01 means significant difference between samples.

The results of PCoA analysis demonstrated that PC1 and PC2 were the main components affecting the microbial community composition between different treatments. PC1 and PC2 contributed 56.11% and 8.09% to the composition of the bacterial community and 36.26% and 7.4% to the composition of the fungal community, respectively (Figure 5). Based on the PcoA results, the soil layer had a greater impact on the community composition of soil microorganisms. The distance between samples of different precipitation treatments decreased with deepening of the soil; that is, the difference between different precipitation events decreased as the soil layer deepened.

### 3.5. Interaction of Dominant Groups of Microorganisms under Treatments

**Figure 6:**
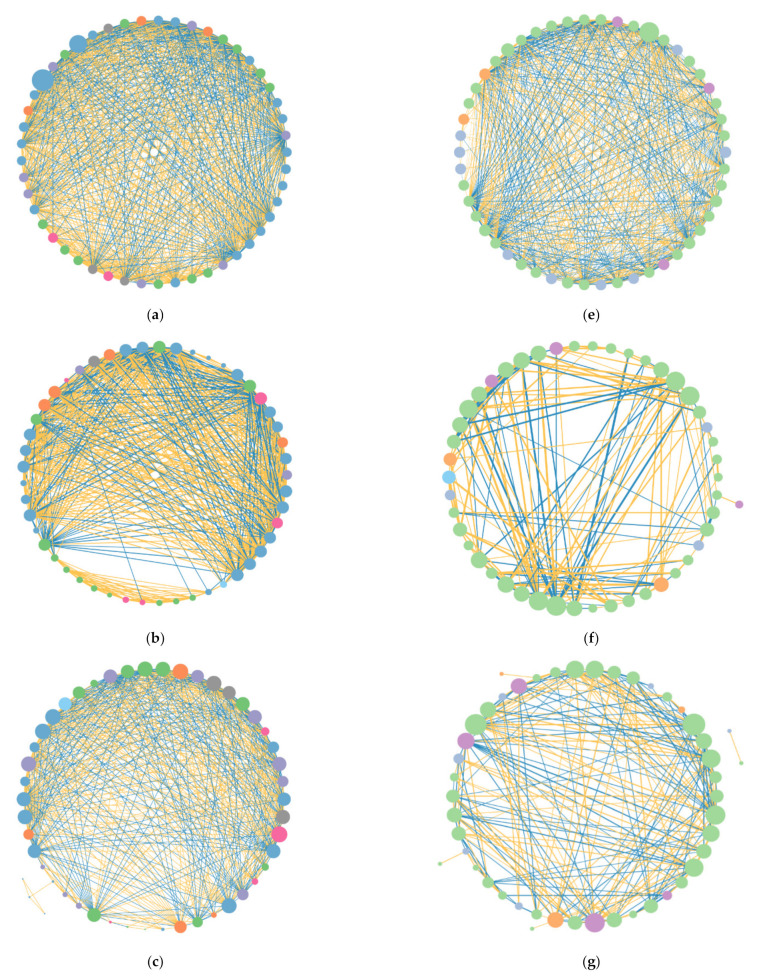

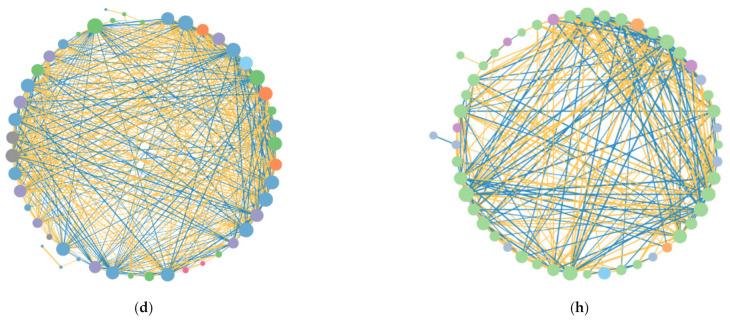
The interactions among soil bacterial (**a**–**d**) and fungal (**e**–**h**) OTUs under different precipitation treatments. (**a**,**e**): the total numbers of soil bacterial/fungal; (**b**,**f**): the numbers of soil bacterial/fungal under P50; (**c**,**g**) the numbers of soil bacterial/fungal under P100; (**d**,**h**) the numbers of soil bacterial/fungal under P150. The node size is proportional to its degree. The line between each pair of nodes represents a strong positive (yellow) or negative (blue) interaction with adjusted *p* < 0.05. The width of the line is the Spearman coefficient size. Only the top 50 most abundant OTUs participated in the analysis.

Network analysis indicated that the number of interactions between bacterial OTUs was 728, of which there were 436 positive interactions and 292 negative interactions; the number of interactions between fungal OTUs was 598, of which there were 300 positive interactions and 298 negative interactions (Figure 6a,b). In general, the correlation between bacterial OTUs is stronger than fungal OTUs.

All networks used the top 50 abundance OTUs for analysis. The total number of links of bacteria was greater than that of fungi, and so were positive links, negative links, average degree, transitivity, clustering coefficient, and network density (Figure 6b–d, Table 3). The network of bacterial was more complicated, connected, and transitive than fungal networks. The total Links and negative links of bacteria decreased under the treatment of increasing and decreasing precipitation. The decline rate of negative links of P50 (28%) was greater than that of P150 (17%) (Table 3). Positive links increased under P50 (13%) and P150 (1%). The networks of bacteria were more sensitive to drought, which promoted the transmission and connectivity of bacterial networks. All links of the fungus had positive feedback to the increase in precipitation gradients (Figure 6e–h, Table 3). The decline rate of negative links of the fungal network was greater than that of positive links in P50, while the increase rate of positive links was greater than that of negative links in P150. Therefore, it can be inferred that increasing precipitation can promote fungus’s cooperation, while reducing precipitation intensified its competition. The transmission and connection of the fungal bacteria network had a negative response to drought.

### 3.6. Relationship between Environmental Factors and Community Composition under Different Treatments

**Figure 7:**
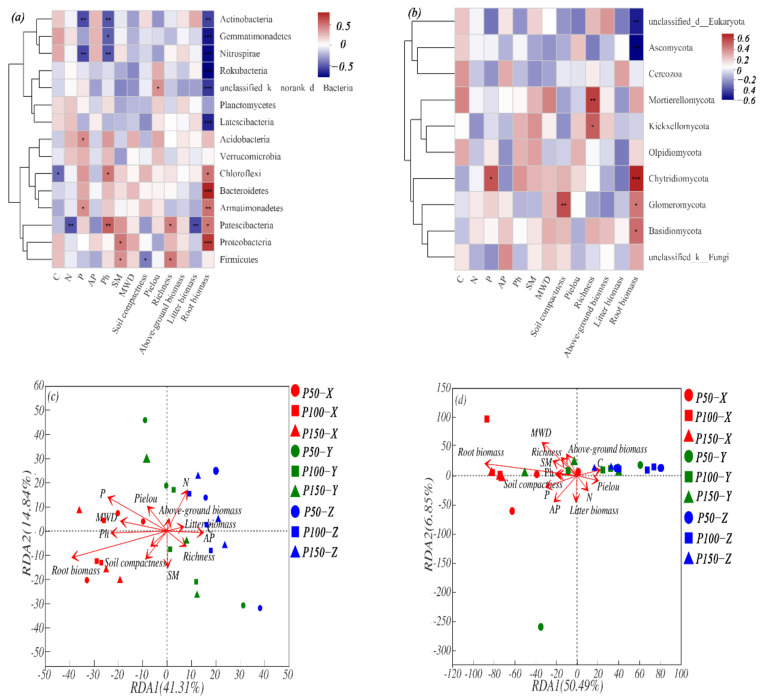
The relationship of the dominant phylum (heatmap) and genera (RDA) of bacteria and fungi with environmental factors. (**a**,**b**): Spearman correlation heatmap of environmental factors with the top 15 bacteria/fungi at the phylum level. The color ratio represents the value of R. (**c**,**d**): Redundancy analysis (RDA) of the dominant genera of bacteria/fungi and environmental factors. *, **, *** indicate *p* < 0.05, *p* <0.01 and *p* <0.001, respectively, among precipitation treatments within soil layers.

A Spearman analysis of the abundance of bacteria and fungi showed that there was a strong correlation between the main bacterial and fungal communities and some environmental factors (Figure 7). Bacterial communities are more affected by environmental factors, including soil and vegetation characteristics, than fungi. The Top15 microbial communities of bacteria and fungi were most affected by root biomass among different environmental factors. Actinobacteria was significantly negatively correlated with *p*, pH and root biomass (*p* < 0.01). Root biomass had a significant negative correlation with bacterial genera such as Gemmatimonadetes, Nitrospirae, Rokubacteria and Latescibacteria (*p* < 0.001), but had significant positive correlation with Chloroflexi, Bacteroidetes, Armatimonadetes, Patescibacteria and Proteobacteria. Root biomass had a significant positive correlation with fungal species such as Chytridiomycota, Glomeromycota, Basidiomycota, but had a significant negative correlation with unclassified_d__Eukaryota and Ascomycota. Redundancy analysis explained 56.15% and 57.34% of the relationship of bacteria/fungi with environmental factors through the top two principal components. RDA also showed that root biomass (*p* < 0.01) and *p* (*p* < 0.05) significantly affected bacterial genera, and MWD and root biomass extremely significantly affected fungal genera (*p* < 0.01).

## 4. Discussion

### 4.1. Response of Microbial Communities to Changes in Precipitation

The proportion of common bacterial and fungal OTUs accounted for 91% and 75% of the total OTUs under different treatments, respectively (Figure 2). The proportion of unique OTUs of fungi under different treatments was greater than that of bacteria, indicating that fungi are more able to adapt to water changes than bacteria [28]. This may be due to the species-specific responses to moisture stress, and arid ecosystems prefer resistant fungi [44]; thus, fungi exhibited more unique OTUs. The amplitude of changes of OTUs showed that 0–10 cm was greater than 20–30 cm under the same precipitation treatment, and the number of OTUs decreased with increasing precipitation under the same soil layer. The number of unique OTUs under P50 was less than that under P150 at 0–10 cm, indicating that surface soil microbes were more susceptible to moisture disturbance, the metabolic activity of the microbial community was reduced with decreasing precipitation, leaving even more to die [44]. The negative reaction of microorganisms to decreased precipitation is manifested as a decrease in the metabolism of the organism [14]. Reduced precipitation causes the soil to dry out, and the fluidity of solutes and enzymes is slower when water shortages and available substrates are scant, which also limits the activities of microbes [44]. Although the number of microbial OTUs changed, the diversity and richness indexes of microorganisms were not significantly different under altered precipitation (Figure 3) [1] and showed significant differences only in the soil layer. The reason may be that precipitation is mostly an intermittent event in arid and semiarid ecosystems, and microorganisms respond strongly to changes in precipitation. Microbial activity at this time occurs throughout most of the year, and even a small amount of precipitation changes can quickly trigger microorganisms to respond [45]. The special rapid growth time of soil microorganisms and the microbial community is likely to change rapidly in a short period of time [15]. However, the study area demonstrated larger amounts of evapotranspiration, which will quickly restore the soil to the control conditions after precipitation events, and the short-term experiment did not change the soil microenvironment. The surface soil is rich in nutrients, plant roots and litter, providing nutrients and other organic matter as available substrates for the metabolism of microorganisms; therefore, the microbial biomass of the surface soil is significantly higher than that in the deep layer.

Variation in soil and vegetation characteristics showed significant effects on the community structure of soil bacteria and fungi [46]. Soil properties like C, N, P, pH, SM, compactness and vegetation characteristics such as richness and root biomass were closely linked to the microbial community structure (Figure 7). Bacterial communities were more likely to fluctuate due to environmental factors than fungal, and various environmental properties were more likely to cause the instability of the bacterial community, rather than the fungal community in fragile grassland ecosystem [46,47].

The dominant microbial communities changed after changes in precipitation, and the explanations for bacteria and fungi under different treatments were 64% and 44%, respectively (Figure 7). In this study, the main indicator groups of bacteria were Actinobacterial, Proteobacterial, Gemmatimonadetes, Rokubacterial, Bacteroidetes, and the main indicator groups of fungi were Ascomycota, Basidiomycota, Mortierellomycota and Glomeromycota (Figure 4). Based on the corresponding patterns of microbial communities in response to precipitation changes, they can be classified into three groups (Figure 5) [1,20]: resource-constrained groups, drought-tolerant groups, and sensitive groups. In this study, bacteria such as Proteobacteria [20] and Bacteroidetes [48] and fungi such as Basidiomycota and Mortierellomycata, which can be explained by trophic theory [13], and can be divided to resource-constrained groups such microorganisms, had an increased chance of obtaining available substrates [13]. Proteobacteria (*p* < 0.01), Bacteroidetes (*p* < 0.01) and Basidiomycota (*p* < 0.05) illustrated significant positive correlation with root biomass. The increasing of precipitation promoted the growth of vegetation and root biomass (Table 1), and the presence of vegetation may increase the available nutrients in the soil and stimulate the increase in the relative abundance of resource-limiting groups. McHugh also pointed out that soil moisture is an important mediator of soil nutrient transport and diffusion [20], and an increase in available water can increase soil dissolution mobility and promote substrate supply [8], while water shortages limit the relative abundance of microorganisms. Second, in the drought-tolerant groups, the relative abundance of microorganisms decreased under increased precipitation but increased under decreased precipitation; that is, the relative abundance of the microbial community of the group increased under water stress and had good drought resistance ability. In our study, the bacteria of Gemmatimonadetes and Rokubacteria and the fungi of Ascomycota also proved to be resistant to drought. They may be affected by the unique physiological characteristics of the microbial communities and the indirect influence of the vegetation or other environmental factors [1]. Bacterial communities have thick peptidoglycans that are connected to each other and have the ability to develop resources in the soil and obtain available water through cell walls [8]. Fungal hyphae also have the function of redistributing and reusing water [1]. Ascomycota is believed to be able to degrade cellulose and other complex carbohydrates; its metabolic capacity is conducive to survival in environments lacking substrates caused by water shortages [49]. In addition, our research has shown that root biomass has a significant negative correlation with those that are microbial. Soil nutrient uptake by roots decreases as root biomass decreases, and the opportunities for microorganisms to obtain nutrients increase [1]. Third is the sensitive group, which had the largest relative abundance in the control, and it decreased with altered precipitation. Such microorganism groups have poor adaptability to the environment and are easily disturbed by soil moisture, such as the bacterial Actinobacteria and the fungi Glomeromycota, which were closely linked to root biomass. Some studies also have indicated that these microbiomes are closely related to the diversity of vegetation communities [50], but our study did not show a strong correlation between microbial communities and vegetation and only showed a trend of increasing precipitation. The reason may be due to the small changes in environmental factors under short-term precipitation treatments, and the evaporation of the study area was much greater than the precipitation, weakening the internal connection between the two. 

### 4.2. Network Relationships Revealed the Bacterial-Fungal Interactions in Different Precipitation Treatments

Microorganisms do not exist in isolation but form a complex network of relationships [27]. The interactions between species and their dynamic changes are crucial in generating microbial responses to climate change [45]. The positive and negative interactions of microbes represent cooperation and competition between the corresponding microbial species [51]. Our research showed that microbial communities differentially respond to drought [47]. Under the changing of precipitation, the network of bacteria was stronger than that of fungi, and the bacterial network was more complex and connected than the fungal network (Figure 6, Table 3); the stronger network of bacteria also had a higher average degree, and de Vries et al. mentioned that ecological networks with weak interactions were more stable than those with strong interactions. Bacterial networks were more sensitive and had poor resistance to drought, and the positive links increased and negative links decreased during drought. At the same time, the transitivity showed the maximum in drought, indicating that the cooperation between bacterial operational taxonomic units (OTUs) intensified and competition weakened in drought and the transitivity had a positive response to positive links. Guan et al. showed that simple network relationships can negatively affect geochemical functions [52]. Thus, the greater complex of the bacterial community in an arid environment is conducive to its response to the adverse environmental conditions of drought. In this study, the cooperation, competition and transitivity between fungi OTUs increased as the precipitation increased. Both the positive and negative links of the fungal network increased with the increasing of precipitation and they had greater changes than the bacteria. This is because the fungi are more resistant than bacteria and have poor adaptability, showing greater flexibility in dealing with drought, that is, greater volatility changes [47].

Our research also considered that the interaction of bacteria and fungi decreased as the soil layer deepened in arid areas, and the positive and negative interactions of bacteria and fungi both increased under altered precipitation on the surface (Table S1). This explained why the interaction of soil bacteria and fungi was affected by precipitation treatments, the surface soil was more sensitive to altered precipitation, and the changes in soil moisture caused by precipitation treatments promoted the strengthening of cooperation and competition interactions among soil microorganisms, complicated the interaction network, and improved the robustness of the microbial interaction network [15].

This kind of cooperation and competition interaction between species is also reflective of their life strategies. The cooperation and competition between microorganisms were conducive to the redistribution of microbial resources and resulted in better survival and metabolism capabilities.

## 5. Conclusions

To study the responses of bacterial and fungal communities and the interaction between microorganisms in response to changing precipitation, we established simulated precipitation devices in semiarid areas. This study showed no significant differences in microbial community diversity among precipitation treatments, but the composition of the microbial community changed with the altered precipitation, and according to the law of its response, it was divided into resource-limited, drought-tolerant and sensitive groups. Compared with fungal networks, soil bacterial networks were less stable but more adaptable under drought. Since microorganisms’ responses to changing precipitation and their interactions are important indicators of ecosystem responses to climate change, and efforts should be continued to identify the feedback mechanism of microorganisms under long-term climate change (precipitation change).

## Figures and Tables

**Table 1:** Variation of vegetation properties under different changes in precipitation (*n* = 3, mean ± SE).

Treatments	Shannon-Wiener	Pielou	Patrick	Coverage	Above-Ground Biomass (g m^−2^)	Litter Biomass (g m^−2^)	Below-Ground Biomass (g m^−2^)
P50	1.46 ± 0.11b	0.88 ± 0.03a	5.33 ± 0.58b	0.65 ± 0.15b	152.74 ± 68.65a	96.41 ± 37.43a	40.54 ± 16.84b
P100	2.35 ± 0.08a	0.84 ± 0.01ab	16.33 ± 1.15a	0.89 ± 0.02a	245.59 ± 49.07a	87.19 ± 32.61a	117.28 ± 25.89a
P150	2.29 ± 0.13a	0.81 ± 0.02b	17 ± 2.65a	0.98 ± 0.01a	276.69 ± 55.95a	88.30 ± 32.51a	56.43 ± 19.95b

Notes: Different lowercase letters indicate statistically significant differences among the different precipitation treatments (*p* < 0.05).

**Table 2:** Variation of soil properties under different changes in precipitation (*n* = 3, mean ± SE).

Treatment	0–10 cm			10–20 cm			20–30 cm			*p* Value from Two-Way ANOVA		
	P50	P100	P150	P50	P100	P150	P50	P100	P150	L	P	L × P
C (g kg^−1^)	16.72 ± 1.34	18.22 ± 1.57	18.76 ± 3.05	13.89 ± 3.81	15.29 ± 1.35	18.07 ± 2.92	14.05 ± 1.70	16.31 ± 2.28	13.80 ± 1.73	0.03	0.18	0.38
N (g kg^−1^)	2.21 ± 0.08	2.12 ± 0.16	2.19 ± 0.05	2.29 ± 0.12	2.24 ± 0.09	2.15 ± 0.09	2.28 ± 0.29	2.10 ± 0.04	1.89 ± 0.25	0.19	0.07	0.35
P (g kg^−1^)	0.74 ± 0.15	0.82 ± 0.00	0.79 ± 0.05	0.85 ± 0.02	0.85 ± 0.03	0.78 ± 0.02	0.83 ± 0.01	0.87 ± 0.03	0.76 ± 0.01	0.28	0.05	0.28
AP (mg kg^−1^)	4.80 ± 1.38	3.68 ± 0.89	4.45 ± 0.32	3.86 ± 1.61	2.85 ± 0.32	3.49 ± 0.79	2.45 ± 0.44	2.49 ± 0.27	2.99 ± 0.95	0.00	0.22	0.79
pH	7.78 ± 0.05	7.84 ± 0.04	7.72 ± 0.08	7.83 ± 0.08	8.02 ± 0.15	7.91 ± 0.08	7.87 ± 0.08	8.06 ± 0.04	7.98 ± 0.22	0.00	0.02	0.63
SM (%)	5.20 ± 0.17	7.29 ± 1.22	8.88 ± 0.10	6.94 ± 1.41	8.71 ± 0.40	8.17 ± 1.49	4.76 ± 0.43	8.64 ± 1.16	9.05 ± 0.38	0.20	0.00	0.046
SFD	2.79 ± 0.03	2.77 ± 0.05	2.78 ± 0.06	2.87 ± 0.05	2.80 ± 0.08	2.75 ± 0.03	2.81 ± 0.05	2.78 ± 0.11	2.82 ± 0.08	0.67	0.37	0.44
MWD	1.73 ± 0.19	1.74 ± 0.37	1.78 ± 0.34	1.24 ± 0.37	1.58 ± 0.65	1.89 ± 0.07	1.71 ± 0.25	1.67 ± 0.52	1.59 ± 0.45	0.63	0.58	0.55
GMD	1.22 ± 0.04	1.22 ± 0.09	1.32 ± 0.08	1.11 ± 0.08	1.19 ± 0.15	1.26 ± 0.02	1.21 ± 0.06	1.21 ± 0.13	1.19 ± 0.11	0.66	0.56	0.56
Compactness (Pa)	1397.30 ± 336.67	705.67 ± 86.16	943.08 ± 331.02	1296.58 ± 610.82	580.92 ± 75.13	914.33 ± 361.56	1466.17 ± 592.27	808.00 ± 189.21	1259.25 ± 384.18	0.39	0.00	0.98

Notes: L, Soil layer; P, Precipitation treatment; L × P, soil layer × Precipitation; C, soil organic carbon; N, soil total nitrogen; P, soil total phosphorus; AP, soil available phosphorus; SM, soil moisture; SFD, Soil fractal dimension; MWD, soil mean weight diameter; GMD, geometer mean diameter. The same as below.

**Table 3:** Network topological features under different precipitation treatments.

Network Topological Features	Bacterial			Fungal		
	P50	P100	P150	P50	P100	P150
Nodes	49	49	48	47	47	50
Total Link	506	535	498	165	233	285
Positive link	332	294	297	100	119	154
Negative link	174	241	201	65	114	131
Average degree	20.653	21.837	20.75	7.021	10.311	11.4
Transitivity	0.865	0.784	0.769	0.497	0.524	0.603
Clustering coefficient	0.813	0.765	0.688	0.461	0.527	0.522
Network Density	0.43	0.455	0.441	0.153	0.234	0.233
Network heterogeneity	0.517	0.482	0.496	0.523	0.511	0.608
Network centralization	0.225	0.264	0.294	0.181	0.254	0.289

## Data Availability

The data presented in this study are openly available in https://doi.org/10.5061/dryad.v41ns1rz8 (accessed on 15 February 2022).

## Supplementary Materials

The following supporting information can be downloaded at: ’https://www.mdpi.com/article/10.3390/microorganisms10040817/s1, Table S1: The interactions among soil bacterial and fungal OTUs under different treatments.
