# Supplementary material for: Responses of Soil Microbial Communities and Networks to Precipitation Change in a Typical Steppe Ecosystem of the Loess Plateau
Source: Microorganisms. 2022 Apr 14;10(4):817. doi: 10.3390/microorganisms10040817 (PMC9027300; doi:10.3390/microorganisms10040817)
Supplement: Supplementary file 1 [file microorganisms-10-00817-s001.zip › microorganisms-1668265-supplementary.pdf]

Table S1. The interactions among soil bacterial and fungal OTUs under different treatments.

Interaction Type		0–10 cm			10–20 cm			20–30 cm		
		**P50**	**P100**	**P150**	**P50**	**P100**	**P150**	**P50**	**P100**	**P150**
Bacterial	Pos	251	185	241	228	205	237	373	217	235
	Neg	242	192	192	238	198	228	228	216	222
	Total	493	377	433	466	403	465	601	433	457
Fungal	Pos	200	185	193	255	170	270	189	186	208
	Neg	164	141	204	75	163	68	190	196	177
	Total	364	326	397	330	333	338	379	382	385
